# Gelation of Soy Milk with Hagfish Exudate Creates a Flocculated and Fibrous Emulsion- and Particle Gel

**DOI:** 10.1371/journal.pone.0147022

**Published:** 2016-01-25

**Authors:** Lukas Böni, Patrick A. Rühs, Erich J. Windhab, Peter Fischer, Simon Kuster

**Affiliations:** 1 Food Process Engineering Group, Institute of Food, Nutrition and Health, ETH Zürich, 8092 Zürich, Switzerland; 2 Complex Materials Group, Department of Materials, ETH Zürich, 8093 Zürich, Switzerland; Massey University, NEW ZEALAND

## Abstract

Hagfish slime is an ultra dilute, elastic and cohesive hydrogel that deploys within milliseconds in cold seawater from a glandularly secreted exudate. The slime is made of long keratin-like fibers and mucin-like glycoproteins that span a network which entraps water and acts as a defense mechanism against predators. Unlike other hydrogels, the slime only confines water physically and is very susceptible to mechanical stress, which makes it unsuitable for many processing operations and potential applications. Despite its huge potential, little work has been done to improve and functionalize the properties of this hydrogel. To address this shortcoming, hagfish exudate was mixed with a soy protein isolate suspension (4% w/v) and with a soy emulsion (commercial soy milk) to form a more stable structure and combine the functionalities of a suspension and emulsion with those of the hydrogel. Hagfish exudate interacted strongly with the soy systems, showing a markedly increased viscoelasticity and water retention. Hagfish mucin was found to induce a depletion and bridging mechanism, which caused the emulsion and suspension to flocculate, making “soy slime”, a cohesive and cold-set emulsion- and particle gel. The flocculation network increases viscoelasticity and substantially contributes to liquid retention by entrapping liquid in the additional confinements between aggregated particles and protein fibers. Because the mucin-induced flocculation resembles the salt- or acid-induced flocculation in tofu curd production, the soy slime was cooked for comparison. The cooked soy slime was similar to conventional cooked tofu, but possessed a long-range cohesiveness from the fibers. The fibrous, cold-set, and curd-like structure of the soy slime represents a novel way for a cold coagulation and fiber incorporation into a suspension or emulsion. This mechanism could be used to efficiently gel functionalized emulsions or produce novel tofu-like structured food products.

## Introduction

**Fig 1:**
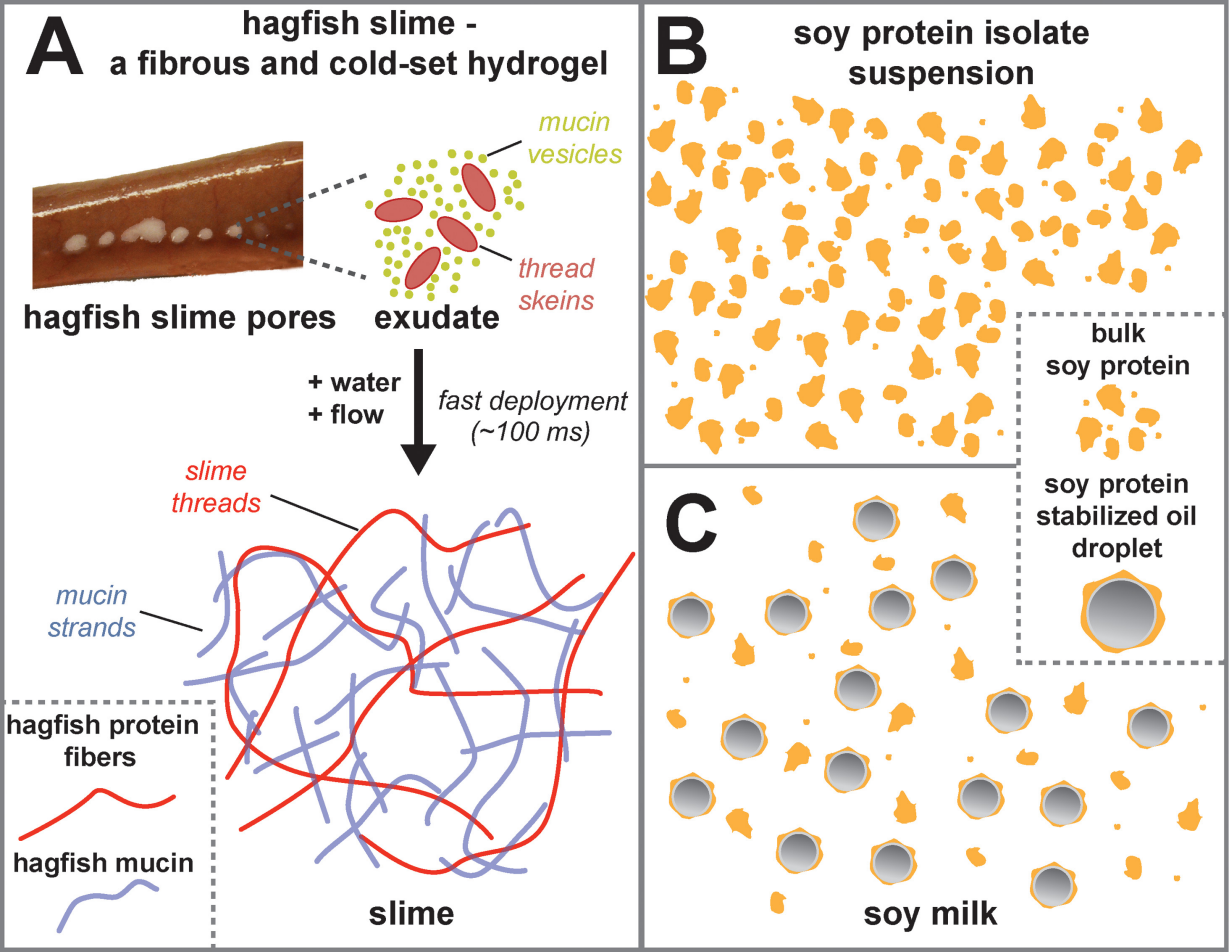
**A** Schematic illustration of the components in hagfish slime. Hagfish exudate is expelled from ventro-lateral pores. Upon contact with water, the thread skeins unravel to long fibers and the vesicles swell, rupture, and mucin strands are formed. The fibers and the mucin together form the slime. **B** Soy protein isolate suspension. **C** Soy milk.

The marine hagfish produces record-breaking amounts of slime when provoked or attacked. The instantaneously formed slime serves as a very effective defense mechanism against predators by clogging their gills [1, 2]. A schematic drawing of the components and the deployment mechanism of hagfish slime is provided in Fig 1A. Hagfish slime is an elastic, tough and coherent soft gel with a complex network structure, consisting of long protein fibers (≈ 15cm) and a hydrated mucus part [1, 3]. The slime is ultra dilute, containing 99.996% water, which is physically confined between the fibers and the mucins and thus, unlike in other hydrogels only transiently retained [4]. The fibers are made of intermediate filaments (IFs) and are phylogenetically related to type II keratins [5]. In their native form they are coiled-coil type of proteins and undergo a so called *α* to *β* transition when subjected to deformation, leading to substantially improved mechanical properties [6]. This *α* to *β* transition appears to be a hallmark of IFs-like proteins [7], imparting hagfish fibers mechanical properties similar to spider silk but exceeding their processing possibilities and bio-physical properties [8].

As hagfish (including their slime) are eaten in Asia (eg. Japan and Korea) [9], their slime is suitable for potential food-grade applications. Although there has been some research with the slime in the recent years, the huge potential of its functional material properties, however, has only been scarcely exploited compared to spider’s silk. Research mainly focused on mimicking the hagfish slime thread on the molecular level [10, 11]. Only minor efforts were made to functionalize and improve this outstanding hydrogel on the meso- and macro-scale [12] regarding structuring, solids incorporation, water retention, and many other features, which could substantially improve its processability and thus make it applicable as a structuring agent.

To investigate structuring effects when hagfish slime is mixed with dispersions and emulsions, the slime is combined with a soy suspension and a soy emulsion. Unlike the transiently retained water, the solids in the suspension and the oils in the emulsion are considered to be better incorporated in the slime matrix during the rapid and cold-set gelation. Thus, novel functional material properties could be imparted, which can be influenced by the suspension and emulsion properties.

To test this hypothesis, soy protein isolate (SPI) suspensions and commercial soy milk were mixed with hagfish exudate. The slime was then rheologically and microscopically investigated regarding its microstructural and flow properties. The components of the soy protein isolate suspension and the soy milk are illustrated Fig 1B and 1C, respectively. Both soy systems were found to be strongly and efficiently gelled and showed markedly improved viscoelastic and liquid retention properties. Moreover, the cold gelation of soy milk resembles the coagulation process during tofu curd production, which in contrast requires heat input, time, and calcium salts as coagulants [13, 14]. In a last part of the manuscript the coagulated system is subjected to a simulated tofu cooking process and compared to a regularly produced tofu.

## Materials and Methods

### 0.1 Exudate sampling and stabilization

The Atlantic hagfish (Myxine glutinosa) were kindly provided by the Atlanterhavsparken in Ålesund, Norway. The sampling was carried out according to the approved ethical application by the Forsøksdyrutvalget (FOTS ID 6912) and followed the protocol of Herr et al. [15] and the advice of Møreforsking Ålesund and was carried out in their laboratory in the Atlanterhavsparken in Ålesund. The hagfish were placed in a 10 l bucket of fresh and cold seawater and anesthetized with a 1:9 mixture of clove bud oil (SAFC) and ethanol (abs.) using a concentration of 1 ml/l. Once unresponsive to touch, the fish were placed on a dissection tray, blotted dry, and electrically stimulated (80 Hz, 8–18 V, HPG1, Velleman Instruments) on the ventro-lateral side. This mild electro-stimulation causes the muscles around the slime glands to contract and the exudate to be released. The exudate was collected with a spatula and put into MCT (medium chain triglycerides, Delios GmbH, Germany) or dispersed in stabilization buffer (0.9 M sodium citrate and 0.1 M PIPES, pH 6.7 solution) [15, 16] and stored at 4°C. Two different stabilization methods were used as either have specific advantages. Exudate stabilized in citrate/PIPES buffer allows a separation of the mucin vesicles from the thread skeins and thus the investigation of the influence of the hagfish mucin on the soy systems alone. However, we found the buffer to diminish the functionality of the whole slime (ie. including the fibers) and therefore used an alternative method to stabilize the exudate (immersed in MCT oil) as already used by Böcker et al. [12]. This stabilization method allows the formation of a highly functional slime, strongly similar to the natural slime but does not allow a separation of the mucin vesicles. A similar stabilization of hagfish exudate under oil for rheological measurements was used by Ewoldt et al. [3]. After the quick sampling, the fish were transferred to a recovery bath. Import and export were granted by the Swiss Federal Food Safety and Veterinary Office (FSVO) and by Norwegian Seafood Council (Norges sjømatråd), respectively.

### 0.2 Shear and oscillatory rheology and sample preparation

For rheological experiments, a shear rheometer (Physica MCR 301, MCR702, Anton Paar, Austria) with a couette geometry (CC27, Anton Paar, Austria) was used. Frequency sweeps were performed at a fixed strain amplitude of *γ* = 1% and amplitude sweeps were carried out at a fixed angular frequency of *ω* = 1 rad/s. For every sample, an amplitude sweep to determine the linear viscoelastic regime and a frequency sweep were carried out. Viscosity measurements were performed using shear rates from 0.1 to 100 s^−1^. All rheological measurements were performed at 20°C.

### 0.3 Slime formation

To prepare the slime for rheological measurements, 4 *μ*l of exudate were placed into a glass flask and 20 ml of milliQ (Millipore), soy milk (Coop, CH; contains 4 wt% protein, 2 wt% carbohydrates, and 2 wt% oil) or 4% w/v soy protein isolate (Loryma, Germany; 90–95 wt% protein) suspension were poured in. The flask was closed and sloshed head over back and forth along the tube axis for eight oscillations until the liquid was gelled and a cohesive slime mass was formed [3]. The content was then transferred to the CC27 cup. An exudate concentration of 0.2 mg/ml was used, assuming an exudate density of *ρ* = 1 g/ml [3]. This is about four times lower than the concentration used by Ewoldt et al. [3] for rheological studies and only about twice as concentrated as natural hagfish slime [4]. A mucin vesicles solution and its mucin content was obtained following the protocol of Salo [17]. Exudate stabilized in citrate/PIPES buffer was filtered through a series (60, 41, and 20 *μ*m) of nylon mesh filters (Merck) to separate the small mucin vesicles from the skeins. To concentrate the vesicles, the filtrate was centrifuged (2000 g, 10 min) and the supernatant was discarded. The mucin content of the solution was determined in triplicates by dialysis (dialysis membrane 25 kD MWCO, SpectraPor, USA) of a defined volume of the stock solution against milliQ water (three batches, 12 hours each) and subsequent freeze drying to determine the dry weight. The mucin concentration of the vesicle solution was 2.6 ± 0.8 mg/ml.

### 0.4 Liquid retention measurements

Liquid retention measurements were performed with an in-house built mixing device attached on top of a laboratory scale (Scaltec, SBC 32). 8 ml of slime mass were prepared according to the protocol above. The flask with slime was placed on the scale, the mixing device lowered into the slime mass and gently revolved ten times to wrap the slime around it. Then the mixing device was lifted, arrested in the upper position, and the liquid egress from the hanging slime was recorded gravimetrically. The change of weight over time was extracted from the recorded video.

### 0.5 Microscopy and electrophoretic mobility measurements

Light microscopy was performed on a Nikon Diaphot (Nikon, Japan) and images were captured with the NIS elements D3.0 software. Liquid samples were placed on microscopy slides with transfer pipettes and covered with a cover slip. Solid tofu-like samples were gently cut from the curd using a scalpel, covered with a glass slip and slightly compressed in order to allow light transmission. The zeta potential *ζ* of hagfish mucin in 1mM citrate/PIPES buffer was determined in triplicates with a Zetasizer (Nano Series, Malvern Instruments, Germany) at 20°C, using a refractive index of 1.450 and an absorption of 0.001. The zeta potential was calculated using the Henry equation and the Smoluchowski approximation $\zeta =\frac{\eta \mu }{\varepsilon_{0}\varepsilon }$, with *η* being the viscosity of the medium, *μ* the electrophoretic mobility, *ε*_0_ the permittivity of vacuum, and *ε* the dielectric constant of the medium.

## 1 Results and Discussion

### 1.1 Interaction of hagfish slime with soy protein

**Fig 2:**
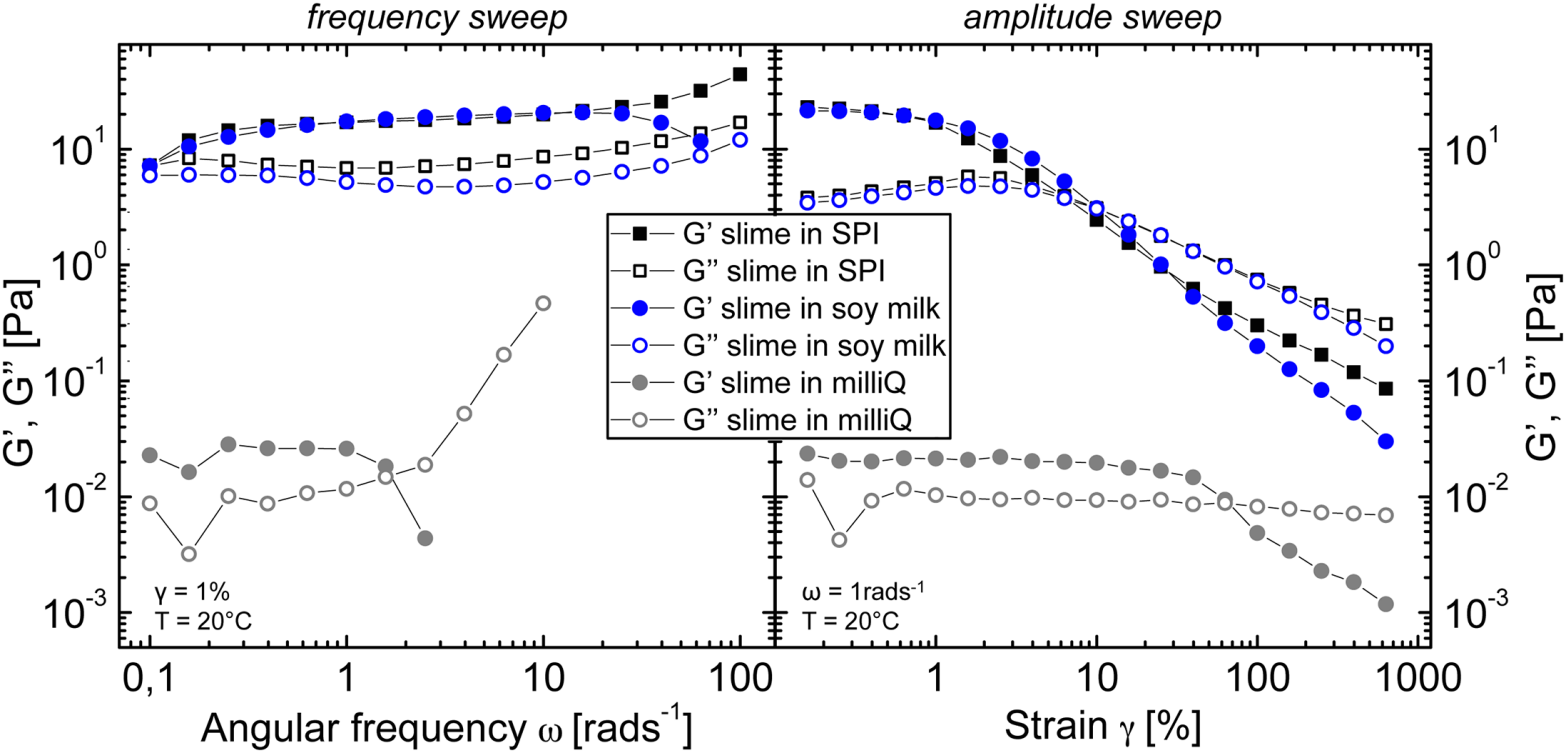
Frequency sweeps (left) and amplitude sweeps (right) of hagfish slime in milliQ, in a 4% w/v soy protein isolate (SPI) suspension (SPI slime), and in commercial soy milk (soy slime).

**Fig 3:**
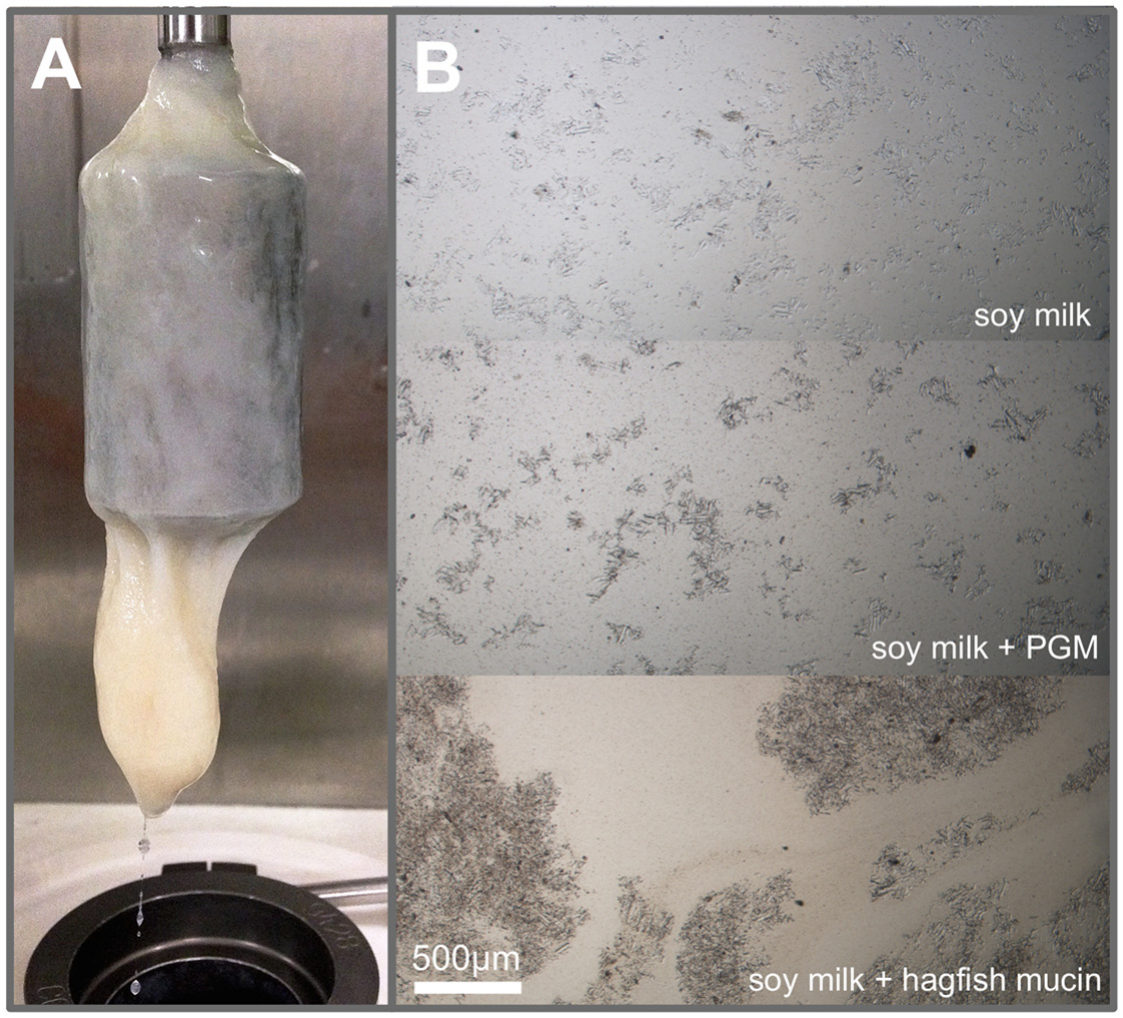
**A** CC27 couette geometry with hagfish slime that was mixed into a 4% w/v soy protein isolate (SPI) suspension. **B** Light microscopy images of soy milk, soy milk with 10 mg/ml porcine gastric mucin (PGM), and soy milk with hagfish mucin (≈ 0.026 mg/ml), showing distinct flocculation.

Mixing hagfish exudate into a soy protein isolate (SPI) suspension was found to create a stronger and more cohesive slime than milliQ or seawater. To quantify this finding, rheological measurements were performed. In Fig 2 frequency sweeps and amplitude sweeps of regular hagfish slime formed with milliQ (grey curve) are compared to hagfish slime formed with a SPI suspension (black curve), hence termed “SPI slime” and to hagfish slime formed with soy milk (blue curve), hence termed “soy slime”. A picture of SPI slime hanging on a CC27 couette after a measurement series is shown in Fig 3A in the following section.

In frequency sweeps hagfish slime showed ultra-soft and weak elastic material properties as already reported by Ewoldt et al. [3], with a strongly increasing loss modulus G” at higher frequencies due to inertia effects and a vanishing elastic modulus G’ at frequencies of 3 rad/s and onwards. In contrast, the moduli of the SPI slime were roughly three orders of magnitudes higher and almost frequency independent. Amplitude sweeps revealed a strain softening behavior for both systems. Natural hagfish slime experienced a softening in G’ but not in G”. The SPI slime strain softened in both G’ and G” and showed a weak strain overshoot in G”, which are characteristics of soft glassy materials such as suspensions and concentrated emulsions [18]. These results suggest a fundamental change in the microstructure for SPI gelled with hagfish exudate.

Very similar rheological results were obtained when hagfish exudate was mixed with commercial soy milk. Soy milk is a more complex and impure system compared to a SPI suspension because additionally to the suspended particles it contains emulsified oil droplets and carbohydrates as it is produced from whole soy meal [13, 14]. The dynamic elastic moduli of the frequency sweep and of the amplitude sweep, however, were almost the same for the SPI slime and the soy slime, suggesting similar changes in microstructure.

### 1.2 Mucin induced flocculation

To determine the factors responsible for the efficient gelation soy milk, the influence of the single components of hagfish exudate on soy milk was investigated. Therefore, in buffer stabilized hagfish mucin vesicles were mixed with soy milk. Fig 3B shows light microscopy images of soy milk, soy milk with hagfish mucin, and soy milk mixed with porcine gastric mucin (PGM, Sigma) as a comparison. Whereas the addition of PGM (10 mg/ml) lead to the formation of small aggregates, the addition of hagfish mucin (≈ 0.026 mg/ml) showed a strong aggregation behavior and the formation of large flocs. Adding stabilization buffer to the soy milk did not cause any visible or rheologically measurable changes. The same aggregation phenomena as with soy milk were observed with SPI and hagfish mucin, with the exception that the trapped particles are larger. Therefore, the aggregation with SPI lead to a phase separation because of sedimentation over time. Flocculation of an emulsion or a suspension creates a class of soft material with distinct viscoelastic properties as a result of structuring [19, 20]. As soy milk contains both, droplets and particles, the network is composed of flocculated emulsion droplets and suspension particles.

The ability of mucins to flocculate emulsions is known and was attributed to the large size of mucin molecules, which can induce a depletion mechanism in emulsions [21]. Bridging, where large polymers adsorb on particles and pull them together [22], is considered another possible mechanism. We therefore infer that the observed flocculation of soy milk with hagfish mucin is caused by a mucin induced depletion and bridging mechanism. The large size of the hagfish mucin strands [23] as well as the negative zeta potential of hagfish mucin (−34.1 ± 13.1 mV, pH 6.7) and of soy protein isolate (−43 mV, pH 6.7 [24]) support the existence of a long range depletion force [25], which occurs between particles and polymers [26]. A simultaneously occurring bridging, which is based on attractive forces, seems likely although the absolute charge of SPI at the tested pH is negative. Soy proteins possess positive charges in the pH region relevant for this study (soy milk with hagfish mucin: pH 6.68) due to histidine and lysine residues, which allow electrostatic attractions necessary for bridging [24].

**Fig 4:**
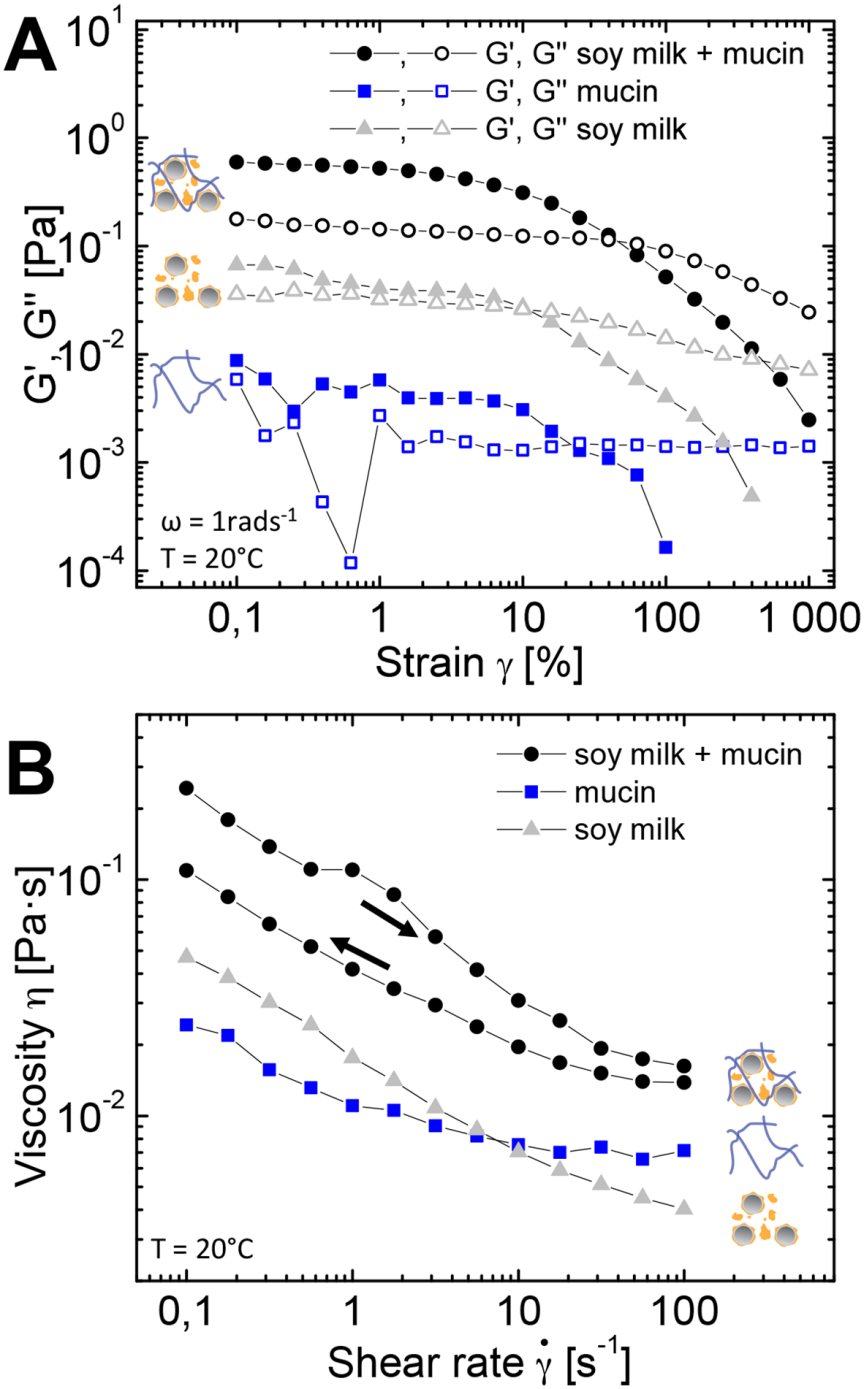
**A** Amplitude sweep and **B** flow curve of hagfish mucin (≈ 0.026 mg/ml) in soy milk and in milliQ. Soy milk + 1% v/v buffer solution and mucin in milliQ are given as a reference. The arrows in **B** indicate the direction of the shear ramp, i.e. from low to high shear rates and vice versa.

A microstructural change and pronounced viscoelastic properties were also seen in rheological measurements, depicted in Fig 4. The amplitude sweep in Fig 4A shows that both, hagfish mucin alone and soy milk alone possessed weak viscoelastic features. Hagfish mucin formed a very weak viscoelastic network at the used concentration (≈ 0.026 mg/ml), which was chosen to imitate the natural mucin concentration (0.02 mg/ml [4]). Soy milk showed a profile typical for an emulsion and suspension system, with a weak elastic response (G’) at low deformations and a clearly increased viscous contribution (G”) compared to water. When the two components were mixed, distinct elastic features developed and the viscous modulus G” jumped up roughly one order of magnitude. The increased viscosity and the hysteresis of the flow curve in Fig 4B further support the occurrence of a structuring by flocculation. The hysteresis in the flow curve is probably caused by a partial break-up of aggregates and thus by a loss of network connectivity at high shear rates.

### 1.3 Mucoadhesive properties of soy lectins

The hagfish mucin flocculated soy milk was only re-dispersible after vigorous stirring, unlike other mucin flocculated emulsions [21, 27]. This inferred the existence of additional attractive interactions. Glycoproteins (such as mucins) are known to strongly interact with lectins, carbohydrate binding (glyco-)proteins, which agglutinate cells and precipitate glycoconjugates [28]. Interactions between glycoconjugates and lectins are crucial to many biological activities [29]. The mucoadhesive properties of lectins [30] together with specific stains are widely used to detect and visualize the highly glycosylated mucins [31]. Despite their low carbohydrate content of 12%, which is far below the content normally found in mucins [17], hagfish mucins are known to bind to lectins [32]. Soybeans contain lectins, which may be still present in SPI [33] despite harsh treatments involving heat, alkali, neutralization, and spray drying. Also, soy lectins are known to specifically interact with N-acetyl-D-galactosamine (GalNAc) [34], which, amongst other lectin binding carbohydrates, is present in hagfish mucin [17]. Based on these literature facts, we propose that soybean lectins bind to hagfish mucin and thus support the observed flocculation. However, further research is needed to test this hypothesis.

### 1.4 Flocculation network enhances liquid retention and viscoelasticity

A prominent feature of hagfish slime is a quick water egress and an irreversible collapse to about 1/50th of its initial volume when lifted up or handled [1], making it rather unapt for mechanical processing. Unlike other hydrogels, that absorb water, most liquid in hagfish slime is only physically retained between the threads. This confinement acts like a very thin-meshed three-dimensional sieve, which transiently holds water [4].

**Fig 5:**
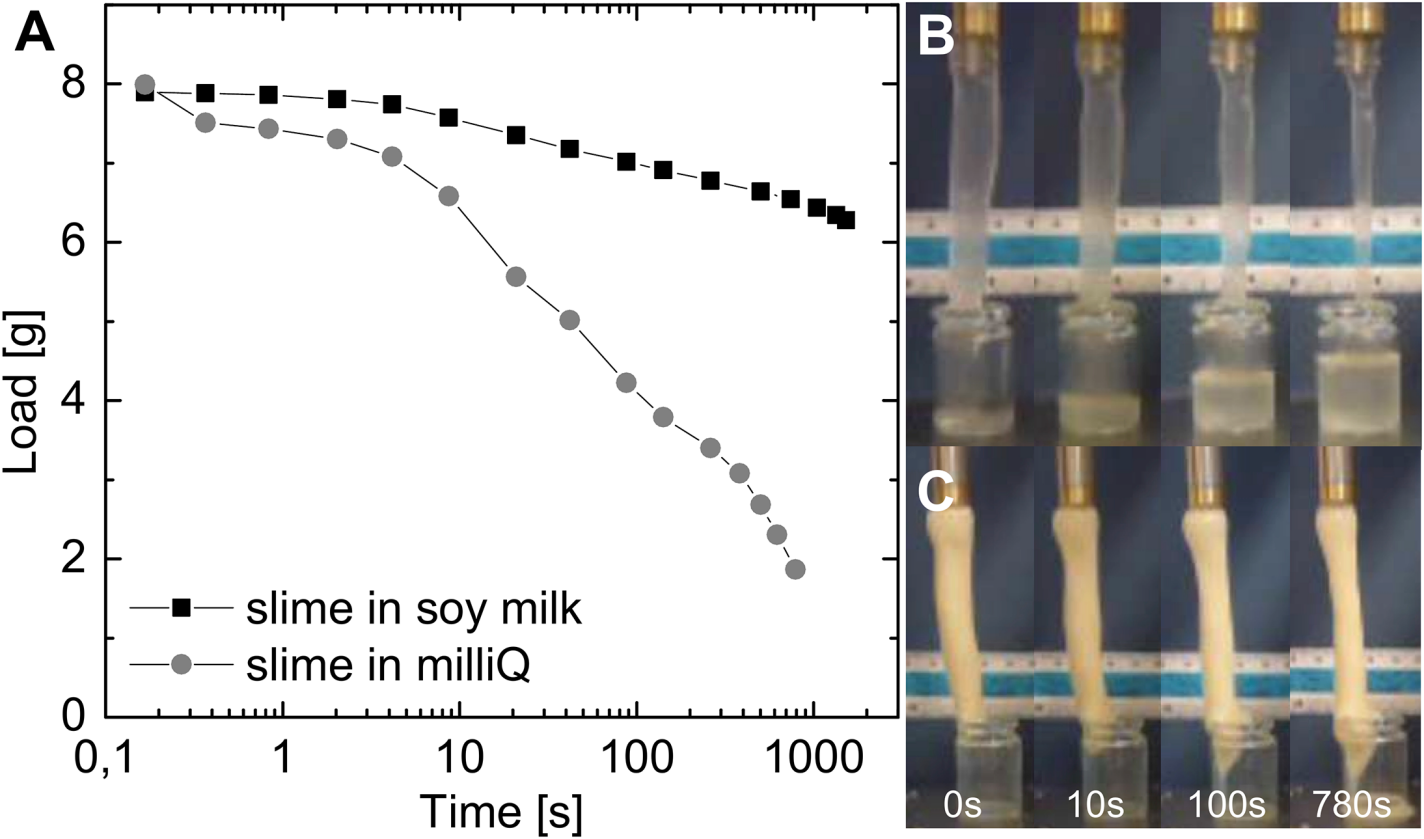
**A** Liquid retention measurements of hagfish slime formed with water and with soy milk. Pictures of the corresponding liquid retention measurements of **B** hagfish slime in milliQ and **C** hagfish slime in soy milk over time (concentration of exudate in milliQ = 4.25 mg/ml, concentration of exudate in soy milk = 1.88 mg/ml).

Mixing hagfish exudate into soy milk not only increased the dynamic moduli in rheological measurements but also showed improved liquid retention properties compared to milliQ as depicted in Fig 5A. Whereas milliQ water drained quickly (Fig 5B), soy slime showed a substantially reduced liquid egress (Fig 5C). Although different amounts of fresh exudate were used (4.25 mg/ml in milliQ, 1.88 mg/ml in soy milk) to gel the liquids, both amounts were sufficient to initially entrap all eight millilitres. After hanging for nearly 30 minutes, however, soy slime still retained most liquid compared to milliQ slime that lost about 3/4 of the initial entrapped water already after the first ten minutes—although more than double the amount of exudate was used. Based on our flocculation theory, the aggregated emulsion droplets and soy particles form a secondary network between the threads. This network supports liquid entrapment in interstitial spaces between the aggregated particles [20] and in additional confinements between the particles and the threads.

**Fig 6:**
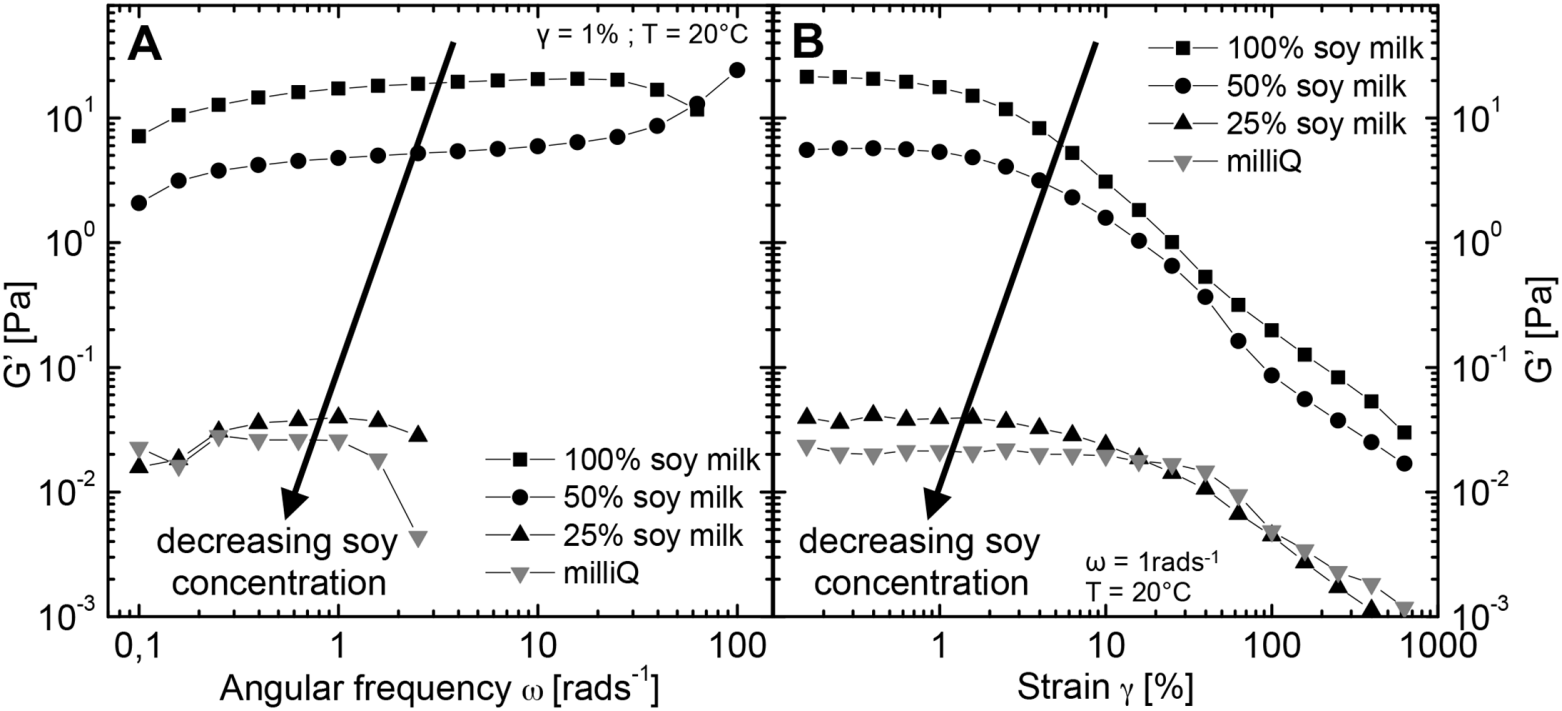
**A** Frequency sweeps and **B** amplitude sweeps depicting G’ of hagfish exudate mixed into a dilution series of soy milk, showing the effect of a decreasing soy concentration on the rheological properties of soy slime.

To determine to what extent the formation of a flocculation network is governed by the soy concentration, soy milk was diluted and subsequently gelled with hagfish exudate. The rheological properties of the soy slime were found to be strongly dependent on the concentration of the soy in the liquid. Fig 6A shows frequency sweeps and Fig 6B amplitude sweeps of a dilution series of soy milk gelled with hagfish exudate, where 100% denotes undiluted and 50% denotes 1:1 diluted soy milk.

An increasing dilution of the soy milk lead to a decreased storage modulus. Whereas the storage modulus at a soy protein concentration of 2% w/v was still increased, further dilution to 1% w/v protein lead to a dramatic loss of elasticity, approaching dynamic moduli of hagfish slime in milliQ. This trend indicates that a minimal amount of soy (2% w/v) in the form of emulsion droplets and particles is needed to establish a flocculated network that contributes to the rheological properties.

### 1.5 Novel fibrous tofu-like products

**Fig 7:**
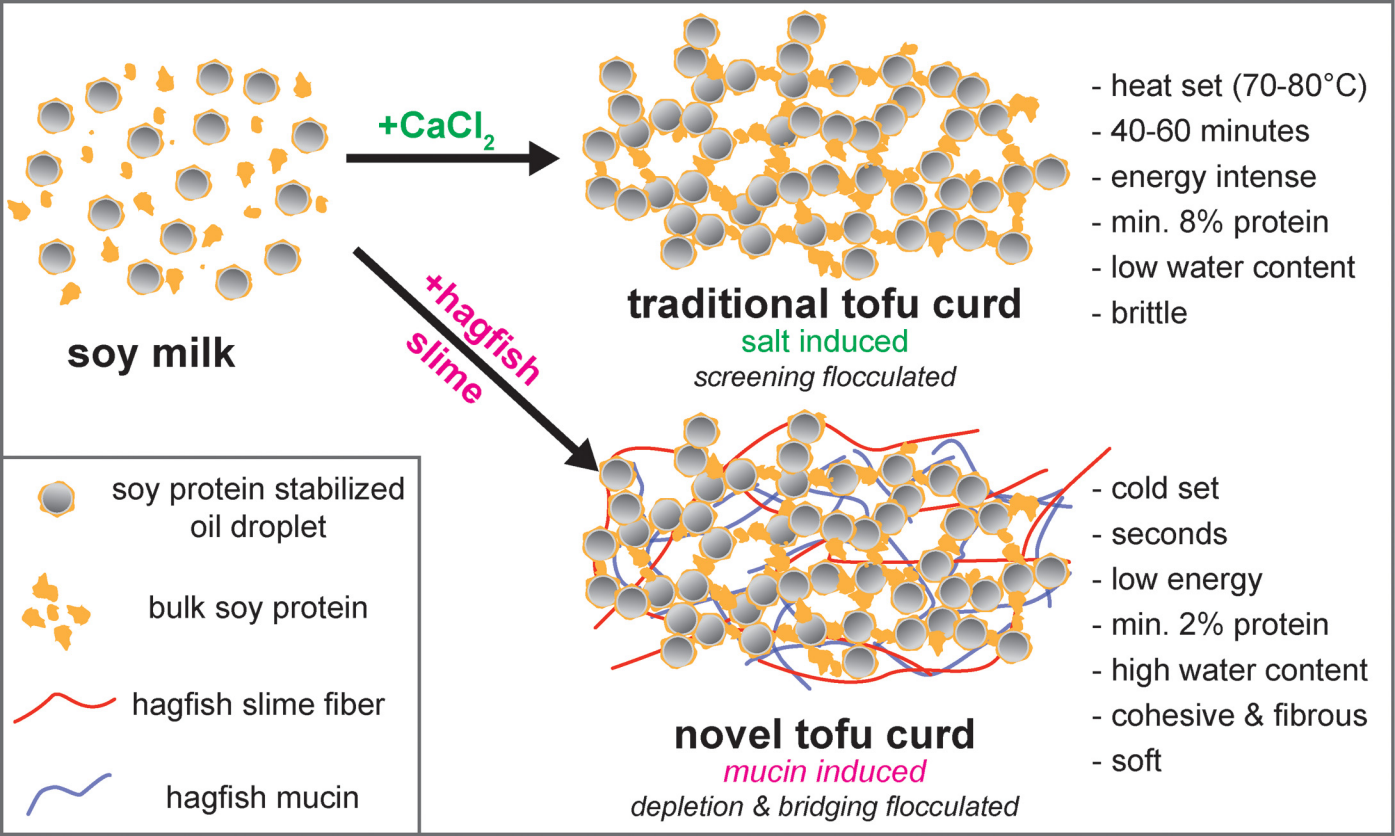
Schematic comparison of a salt induced flocculation of soy milk used in traditional tofu manufacture to the hagfish mucin induced flocculation.

The aggregation when hagfish exudate is mixed into soy milk resembles an acid or salt (Ca^2+^ or Mg^2+^ salts) induced curd formation in traditional tofu production [14]. A schematic illustration of both coagulation processes is shown in Fig 7. To identify the potential of soy slime as a novel tofu-like structured product, a tofu cooking process was simulated in a rheometer and the properties of soy slime were compared to those of regular tofu.

**Fig 8:**
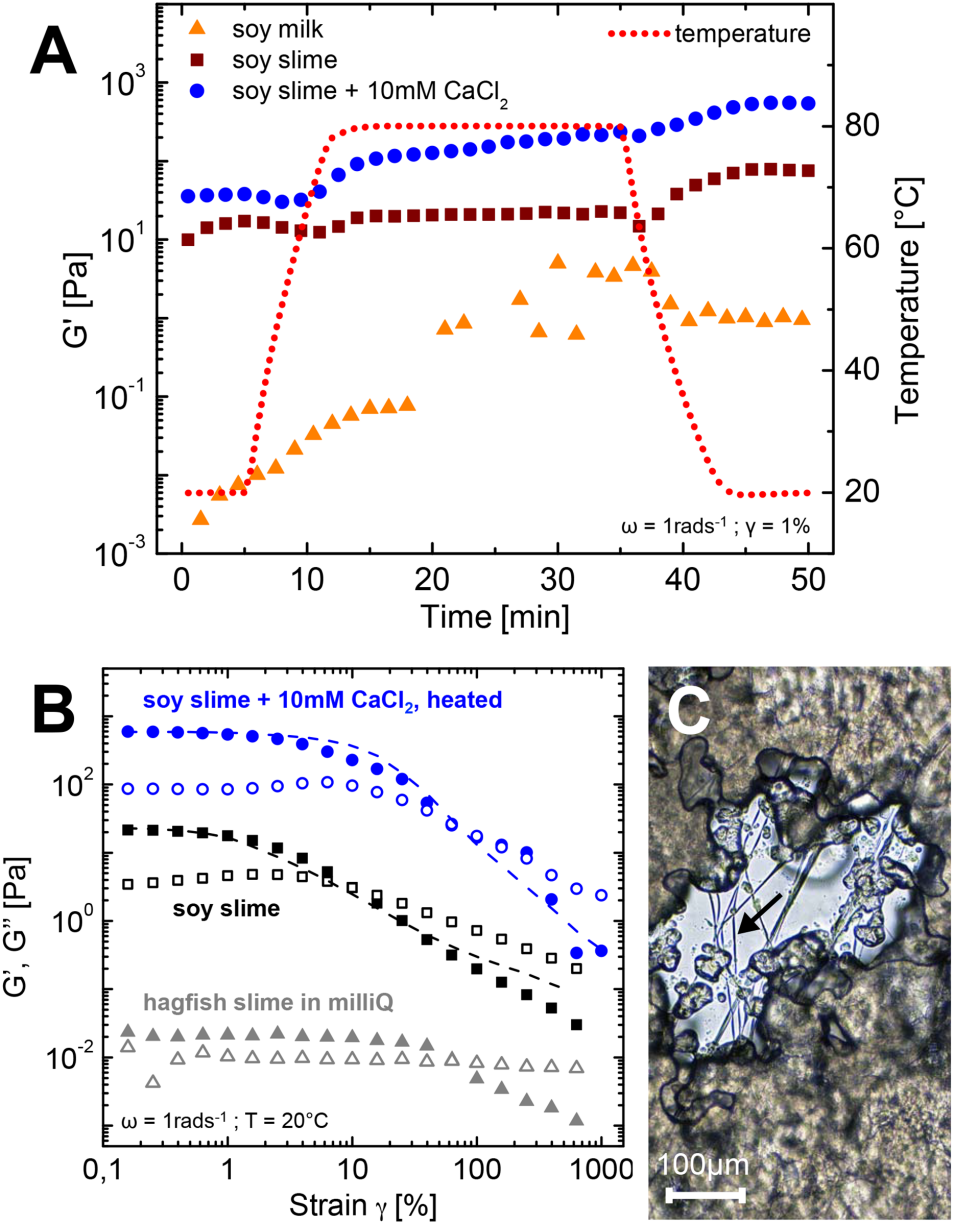
**A** Time temperature sweep as a tofu cooking simulation of soy milk, slime in soy milk and slime in soy milk + 10 mM CaCl_2_. **B** Amplitude sweeps of soy slime + 10 mM CaCl_2_ after the time temperature measurement (blue), uncooked soy slime (black) and hagfish slime in milliQ (grey). The overlaid dotted lines denote G’ of the corresponding measurements for soy protein isolate (SPI) suspensions. **C** Light microscopy image of a cooked 4% w/v soy protein isolate (SPI) suspension + 10 mM CaCl_2_ gelled with hagfish exudate (after the time-temperature sweep). The arrowhead depicts a structuring hagfish slime fiber embedded in the gelled soy matrix.

Traditional tofu production comprises three steps: (1) production of soy milk, (2) coagulation, and (3) optional pressing [35]. Silken tofu, a variety of traditional tofu, lacks the final pressing step because the soy milk coagulates as a whole, which prevents whey separation after adding the coagulants [13]. Soy slime resembles silken tofu as it also possesses a soft and smooth texture and shows no separation of whey after coagulation. In contrast to silken tofu that requires about 10% w/v soy protein [13], the coagulation with hagfish exudate works at soy protein levels as low as 2% w/v. A rheological simulation of a tofu cooking process is shown in Fig 8A. Time-temperature sweeps in small deformation oscillatory measurements are an established method to study gelation processes of biopolymers [36] and an experimental protocol designed to investigate the gelation processes of glycinin and *β*-conglycinin solutions from soybeans was used [37]. During heating all samples underwent gelation, which can be seen in the strong increase of the storage moduli. In contrast to the preceding random aggregation of the coagulation process, the heat induced gelation designates a more ordered re-association of the preliminary partially unfolded proteins [38]. The weakest increase in G’ was observed for pure soy milk caused by the low soy concentration and the lack of divalent ions (Ca^2+^ or Mg^2+^) to form salt bridges between protein aggregates and shield negative charges [36, 39] Therefore, no real network structure could be formed. The soy slime showed a more pronounced gelation behavior. Although there were no divalent cations present, the mucin induced flocculation allowed a good cross-linking between the soy particles.

The soy slime with calcium chloride showed the highest initial storage modulus and also experienced the strongest increase in G’ during heating. The initially higher modulus and the stronger increase of the modulus during heating are caused by calcium salt bridges between protein aggregates and shielded negative charges. These effects allow a closer jamming and a facilitated network formation of the soy particles, resulting in a higher elasticity [36, 39]. Also, mucins are known to interact with calcium ions by forming reversible cross-links and large assemblies [40], which also results in an increased elasticity [41]. During cooling the differences in G’ between soy slime and the soy slime with CaCl_2_ stayed almost constant, inferring similar interactions of the re-associating polypeptides (hydrophobic associations, hydrogen bonding, ionic interactions [38]) that eventually form a strong gel.

The amplitude sweeps in Fig 8B show a summary of the findings of the tofu cooking process and compare the cooked with the uncooked systems. The fine dashed lines represent the storage modulus G’ for the corresponding experiments with SPI instead of soy milk and were added to emphasize the similarity of the experiments. The blue curve depicts the heated soy slime with CaCl_2_. It showed distinct features of gelation, such as an extended linear viscoelastic (LVE) regime and a strain softening at higher strains. In contrast, the unheated soy slime displayed a comparably short LVE regime and a weak strain overshoot in G” as stated above. The fibers, however are present in all the systems and contribute to the cohesiveness, extensibility, and structuring as depicted in Fig 8C. These results demonstrate that combining hagfish slime with soy milk, CaCl_2_, and heat transforms natural hagfish slime into an almost five orders of magnitudes stronger, heat-set, and fibrous gel with a cross-linked continuous matrix.

Soy proteins are known for a high oil holding capacity during cooking. While most animal meats leak fat when cooked in hot water, tofu-curd barely loses oil [36]. The high oil holding capacity of soy proteins in combination with a cold and quick coagulation of the system with hagfish slime could give rise to novel processing possibilities, such as adding sensitive functional ingredients to the oil phase, which is emulsified with soy protein. A subsequent flocculation of the emulsion with hagfish slime would then create a cohesive functionalized curd that would not leak the functional ingredient during cooking, which was already similarly shown for riboflavin [42].

## 2 Conclusion

Hagfish exudate was found to form a strongly and efficiently gelled slime with improved viscoelastic and liquid retention properties when mixed into a soy protein isolate suspension or into soy milk. The additional structuring originates in the formation of a flocculation network. The network is created by a hagfish mucin induced depletion and bridging flocculation of the soy particles and soy protein stabilized emulsion droplets. Additionally, soy lectins are likely to interact with hagfish mucin and thus support the flocculation network. The increased viscoelasticity of the soy slime was found to be dependent on soy protein concentration in the soy milk. A soy concentration between 1–2% w/v in the soy milk was necessary for the flocculation properties to be measurable.

Flocculated soy milk represents a cold-set emulsion- and particle gel, representing a specific type of soft glassy material. The so called soy slime combines increased viscosity and elasticity of soft glassy materials with the cohesiveness and structuring of fibrous hagfish slime. The flocculation network substantially contributes to liquid retention by entrapping more liquid more efficiently in the newly formed confinements between aggregated particles and fibers. Furthermore, the flocculation network provides stability and prevents a rapid collapse of the fiber network under mechanical stress.

The fibrous, cold set, curd-like structure of soy slime is similar to an acid or calcium-salt induced tofu coagulum. The heat gelled product resembles cooked tofu but possesses a long-range cohesiveness provided by the hagfish fibers. Like in industrial tofu production, the addition of 10 mM CaCl_2_ resulted in higher dynamic viscoelastic moduli given a better cross-linking of the soy proteins. The cold-coagulation and fiber incorporation into an emulsion could help to efficiently immobilize heat sensitive nutrients in emulsion systems or produce novel tofu-like structured food products.
